# Assessment of health-related quality of life and patient’s knowledge in chronic non-specific low back pain

**DOI:** 10.1186/s12889-020-09506-7

**Published:** 2021-04-23

**Authors:** Melinda Járomi, Brigitta Szilágyi, Anita Velényi, Eleonóra Leidecker, Bence László Raposa, Márta Hock, Petra Baumann, Pongrác Ács, Alexandra Makai

**Affiliations:** 1 Institute of Physiotherapy and Sports Science, Faculty of Health Sciences, University of Pécs, Vörösmarty u. 3., Pécs, H-7621 Hungary; 2 Department of Neurosurgery, Clinical Center, University of Pécs, Rét utca 2, Pécs, H-7623 Hungary; 3 Faculty of Health Sciences, University of Pécs, Vörösmarty u. 4, Pécs, H-7621 Hungary

**Keywords:** Low back pain, Health-related quality of life, Short form health survey, Roland Morris disability questionnaire, Social determinants

## Abstract

**Background:**

Chronic non-specific low back pain syndrome (cnsLBP) is a severe health problem in developed countries, which has an important effect on patients’ quality of life and is highly determined by socio-demographic factors and low back pain specific knowledge. We examined patients’ health-related quality of life according to the results of the Short Form Health Survey (SF-36), low back pain knowledge (LBPKQ) and the social determinants of the participants.

**Methods:**

We carried out our research in the first half of 2015 in Southern Transdanubia, Hungary. The examination included 1155 respondents living with chronic non-specific low back pain. The confidence interval of 95% was used, and the level of.

significance was *p* < 0.05 using SPSS 22.0 software.

**Results:**

The SF-36 questionnaire is suitable for the examination of patients’ health-related quality of life (Cronbach’s Alpha> 0.76), as the LBPKQ’s Cronbach’s Alpha was 0.726 also, which showed good validity. Longer-term disease meant a lower health-related quality of life (*p* < 0.05). A greater decrease of function (Roland Morris scores (RM)) accounts for a lower HRQoL and higher knowledge level. We found significant differences in LBPKQ scores according to sociodemographic parameters. The general health status was positively correlated with LBPKQ (*p* = 0.024) adjusted for demographic and pain and functional status.

**Conclusion:**

The negative effect of the symptoms on patients’ quality of life is proved, which is determined by different socio-demographic parameters furthermore by knowledge. Above all could be useful information for professionals to adopt the right interventions.

## Background

Chronic non-specific low back pain syndrome (cnsLBP) is a severe health problem in developed countries. Low back pain (LBP) syndrome can be classified according to the duration of the pain as well as the causes triggering the pain. Based on the duration of the pain it can be acute, subacute or chronic. In the case of acute LBP pain lasts for up to 6 weeks, in case of subacute LBP lumbosacral pain lasts from 6 to 12 weeks. Having chronic LBP pain stays for more than 12 weeks. In the case of non-specific LBP diagnosis, a clarified cause can be defined only in 15% of the cases [1–4].

Prevalence of chronic LBP (cnsLBP) among adults is 60% [1, 5]. Prevalence of cnsLBP is strongly associated with several socio-demographic determinants: it is increased by age, being the most characteristic musculoskeletal disorder among middle-aged adults. Women are more commonly affected as well as people performing sedentary occupations or hard physical work including heavy-weight lifting [6–8]. Moreover, obesity can be named as a risk factor for cnsLBP syndrome [5].

Furthermore, the effect of the disease is notable for social and economic subsystems also because in developed societies cnsLBP is the most common reason for a leave of absence from work [9]. The financial burden caused by cnsLBP syndrome has a significant effect on the financing of our healthcare system in Hungary, too [10–16].

Waxman et al. examined the correlation between cnsLBP and symptoms of depression. Pain encourages the patient to tackle the disease, influences the quality of life, and decreases life satisfaction [9, 10].

Nevertheless, studies on quality of life examine the indirect expenses of cnsLBP from the point of view of the individual and also their impact on subjective quality of life, in terms of how much the average of the patients’ HRQoL dimensions differs from the one of healthy population or how much it changes after certain treatments had been applied [17–19].

From the 1990s on the number of studies about quality of life has multiplied in the field of medical-sanitary research; Veresciagina et al. as well as studies of Dahl et al. proved that 36-Item Short Form Health Survey (SF-36) questionnaire is suitable to measure cnsLBP patients’ quality of life [18, 19].

Patients do not have adequate knowledge on the quantity of physical activity and the type of physical activity that can be performed in cnsLBP [20, 21]. Also, patients have little information on how to replace some of the possibly painful activities with other movements in a different way. For healthy and patients with LBP, patient education programs or back school programs can help prevent and rehabilitate spine prevention knowledge or disease specific knowledge [22].

In many diseases, prevention and rehabilitation programs supplemented with patient education are more effective. A patient with a better disease specific knowledge, who understands the cause, pathomechanism of the disease, has the right knowledge of prevention and treatment options, can participate more effectively and actively in prevention or rehabilitation. Thus, patient education is an essential part of primary prevention, to prevent the disease, secondary prevention to reduce the impact of the disease, and tertiary prevention to soften the impact of the illness [23–26].

Weckbach et al. examined 248 patients low back pain knowledge, where they found that only 32.6% of them could respond correctly to the knowledge scale questions [25]. Patients do not have adequate knowledge on the quantity of physical activity and the type of physical activity that can be performed in cnsLBP [20, 21]. Also, patients have little information on how to replace some of the possibly painful activities with other movements in a different way. For healthy and patients with LBP, patient education programs or back school programs can help prevent and rehabilitate spine prevention knowledge or disease specific knowledge [22].

One of the objectives of our research is to assess the quality of life (HRQoL) of nscLBP syndrome patients and the functional status of the spine, testing the validity of the HRQoL scale and comparing the scales with healthy adults’s mean values. We hypothesized that the socio-demographic parameters, self-rated physical disability (RMDQ - Roland Morris Disability Questionnaire) and low back pain specific knowledge (LBPKQ) would determine significantly the HRQoL measures by SF-36of nscLBP patients. This study aims help the better understanding of the disease and encourage health care workers to considering socio-demographic and knowledge factors also in planning interventions.

## Methods

### Study design and setting

Patients with cnsLBP (participating in physiotherapy treatment) were recruited into a cross-sectional, observational study between 1 January – 15 July 2015 in Southern Transdanubia, Hungary.

The study based on a convenience sample. Inclusion criteria: patient treated in the Southern Transdanubian region, of age over 18, with chronic ongoing for at least 12 weeks) non-specific low back pain. Exclusion criteria: acute and subacute low back pain syndrome, low back pain with non-musculoskeletal origins (gynecological, retroperitoneal, abdominal), spine surgery in the preceding 6 months, chronic pain syndrome, depression.

The method of our research was a paper and pencil questionnaire that furthermore contained an information sheet about the details of the research and ethical information stating that by filling in the questionnaire the respondent agrees to take part in the study.

This study followed the principles of the Declaration of Helsinki, ethics approval number: ETT TUKEB 5739/2015).

### Measures

The present research consisted of three validated standard questionnaires and a demographic questionnaire designed by our team.

#### Quality of life

The 36-Item Short Form Health Survey (SF-36) [27] was used to measure patients’ HRQoL, which is adapted for respondents over the age of 14 and validated in Hungary [28]. The summary scores of the different items can be derived (range: 0–100) with higher scores indicating better HRQoL. Quality of life is evaluated according to eight different dimensions: Physical functioning (PF), Physical role functioning (RP), Bodily pain (BP), General health (GH), Vitality (VT), Social role functioning (SF), Emotional role functioning (RE), Mental Health (MH).

#### Disease-specific knowledge

The assessment of spine prevention and low back pain disease-specific knowledge was performed with validated Low back pain knowledge questionnaire [24]. The questionnaire consists of 16 single or multiple-choice questions. The questions can be divided into three categories: (1) general knowledge about lower back pain, spinal anatomy and biomechanics, (2) definitions related to lower back pain, (3) prevention and treatment of lower back pain. The maximum score of the questionnaire is 24 points [24, 26].

#### Spine functional state

Roland Morris Disability Questionnaire (RMDQ) was used to measure the functional status of the spine. The 24-item RMDQ can be applied in the cases of acute, subacute and chronic low back pain patients to measure the functional status and the decreased function of the lumbar spine during daily physical activity. The questionnaire has been validated in Hungary, its maximum score being 24 that indicates decreased function/restraint, value 0 implies the maximum [29, 30].

### Statistical analysis

Descriptive statistics were used to summarize demographic parameters. The continuous variables were analysed by using non-parametric statistical tests (Kruskal-Wallis test, the Mann-Whitney U test and Sperman’s rank correlation). Multiple regression analysis was performed using a linear regression model to identify the determinants of HRQoL for the SF-36. Cronbach’s alpha was used to measure the instruments’ internal consistency. SPSS 24.0 was used for all data analyses and all *p*-values < 0.05 were indicated as being of a statistical significance adjusting also for multiple comparisons.

## Results

Patients with cnsLBP were recruited into our cross-sectional study, through selection 1500 forms were planned to be included in our research; after falling-off (due to rejection or exclusion because of inadequate filling in) 1155 participants (439 female, 716 male) were included. Their mean age was 45.25 ± 16.90.

Among 1155 participating cnsLBP patients the ratio of women was 61.99%, two-thirds of the respondents were town residents, 46.03% of them were married. Based on their age (*p* = 0.17) and place of residence (*p* = 0.35) no significant difference was found between genders. According to their marital status, the ratio of the married was significantly higher among men (50.93%), the ratio of the widow was higher among women (9.99%). 65.89% possessed secondary level qualification, among men the ratio of those having a lower qualification is significantly lower (*p* < 0.01). 67.53% are active employees (among women the ratio of the inactive is significantly higher: 35.99% (p < 0.01), 56.45% of respondents do sedentary work.

They first experienced the symptoms of cnsLBP syndrome 15.28 ± 12.55 years ago on the average. To RMDQ, measuring the functional status of the spine, respondents gave the average value of 4.81 ± 4.59, a significant difference between genders was not found (Table 1).

**Table 1:** The socio-demographic characteristics of cnsLBP patients

		Total sample (***N*** = 1157)	Female (61.99%)	Male (38.01%)	Differences between gender (p)
**Age (standard deviation (SD))**		45.25 ± 16.90	45.89 ± 17.34	44.16 ± 16.10	0.17
**Place of living**	county town	28.41%	30.29%	25.49%	0.35
	city	42.34%	40.63%	45.10%	
	village	29.25%	29.08%	29.41%	
**Marital status**	single	22.18%	21.38%	23.61%	< 0.01
	married	46.03%	43.04%	50.93%	
	cohabitated	15.63%	15.61%	15.74%	
	divorced	9.17%	9.99%	7.64%	
	widow	6.99%	9.99%	2.08%	
**Education**	low	5.71%	7.00%	3.40%	< 0.01
	medium	65.89%	49.40%	58.00%	
	high	28.40%	43.60%	38.60%	
**Employment status**	employed	67.53%	64.01%	74.20%	< 0.01
	other - inactive (student. Retired etc.)	32.47%	35.99%	25.80%	
**Employment form**	sedentary work less that 30 min physical activity	24.10%	28.06%	18.77%	< 0.01
	sedentary work with more than 30 min physical activity	32.35%	37.19%	25.85%	
	light physical labour	30.41%	26.73%	35.38%	
	heavy physical labour	13.14%	8.02%	20.00%	
**RMDQ** (maximum 24 point) – mean (SD)		4.81 ± 4.59	4.81 ± 4.54	4.80 ± 4.68	0.63
**Time spent after the first episode of cnsLBP (years)**		15.28 ± 12.55	15.48 ± 12.82	15.27 ± 12.52	0.65

The reliability of the SF-36 questionnaire was examined by testing the Cronbach’s Alpha values of the 8 dimensions, based on the values above 0.76 the internal consistency of the questionnaire is considered acceptable.

Respondents evaluated all HRQoL dimensions with significantly lower scores compared to the healthy population (except for Social functioning (SF) dimension that resulted in lower value but showing no significant difference (*p* = 0.24) [28]. According to the results of SF-36 questionnaire respondents evaluated their general health (GH) with an average of 55.69 ± 20,86 scores out of 100 (which score indicates excellent health conditions). The lowest evaluation was given to the VT dimension (53.45 ± 19.91), indicating low levels of vitality and vivaciousness of nscLBP patients. The measure of BP dimension among nscLBP patients was, as expected, unfavourable (64.24 ± 23.07). Restraints originating from their disease were present during work as well (PR:63.94 ± 38.71) (Table 2).

**Table 2:** Reliability and internal consistency of the different SF-36 dimensions (Cronbach’s Alpha) [29]

	Healthy population - normal values [25]	cnsLBP patients		
		Mean	Standard deviation	p
**General health (GH)**	64	**55.69**	20.86	< 0.01
**Bodily pain (BP)**	78	**64.24**	23.07	< 0.01
**Social function (SF)**	80	**76.86**	23.77	0.24
**Mental health (MH**	71	**67.34**	19.38	< 0.01
**Vitality (VT)**	70	**53.46**	19.91	< 0.01
**Role emotional (RE)**	78	**73.69**	37.24	0.02
**Role physical (RP)**	79	**63.94**	38.71	< 0.01
**Physical function (PF)**	91	**74.67**	23.19	< 0.01

The validity of the LBPKQ was examined, the Cronbach’s Alpha = 0.72. The mean LBPKQ score was 7.81 ± 2.68. We tested the LBPKQ score differences according to different socio-demographic parameters and we found significant differences in scores by age, education, place of living, work type and marital status also (Table 3).

**Table 3:** LBPKQ scores mean according to the socio-demographic parameters

LBPKQ scores				
		Mean	SD	p
**Age groups**	18–29	8.29	2.64	< 0.01
	30–39	7.69	2.78	
	40–49	7.86	2.52	
	50–59	7.79	2.69	
	60+	7.26	2.71	
**Gender**	female	7.91	2.74	0.09
	male	7.66	2.58	
**Education**	low	6.20	2.35	< 0.01
	middle	7.70	2.63	
	high	8.39	2.70	
**Place of living**	urban	7.98	2.65	< 0.01
	rural	7.38	2.69	
**Having a paid work**	yes	7.89	2.62	0.15
	no	7.67	2.73	
**Work type**	sitting, less than 30 min activities	8.10	2.57	0.03
	sitting, more than 30 min activities	8.22	2.69	
	light work	7.67	2.58	
	heavy work	7.39	2.53	
**Marital status**	single	8.19	2.65	< 0.01
	cohabitating	7.79	2.63	
	married	8.07	2.68	
	divorced	7.30	2.51	
	widow	6.98	2.86	

In the course of our examination we assumed that the increase of the years spent since the first episode of low-back pain implies a further decrease in the quality of life of cnsLBP patients. Regarding all 8 dimensions of the questionnaire significant correlation was found between the number of years spent in pain and the mean value of the certain HRQoL dimension (p < 0.05).

Among the examined scores weak correlation was found (R < 0.30), except for PF that showed medium strength correlation with the number of years, indicating significantly decreased function by the ongoing of years (R = -0.41; *p* < 0.01) (Table 4).

**Table 4:** Correlations between the time spent since the first episode of LBP, RM, LBPKQ and HRQoL

	Diagnosed	RM	LBPKQ
**General health (GH)**	−0.27*	− 0.4*	0.11*
**Bodily pain (BP)**	−0.29*	−0.59*	0.06*
**Social function (SF)**	−0.17*	−0.46*	0.07*
**Mental health (MH)**	−0.06*	−0.29*	0.04**
**Vitality (VT)**	−0.14*	−0.39*	− 0.02**
**Role emotional (RE)**	−0.09*	− 0.27*	0.02**
**Role of physical health (RP)**	−0.23*	−0.52*	0.08*
**Role of physical functioning (RP)**	−0.41*	−0.58*	0.04**

p < 0.05 *; *p* > 0.05 **

Results of RMDQ was significantly correlated with all 8 HRQoL dimensions (p < 0.01) (showing negative weak or medium strength correlation) (Table 4).

LBPKQ was showed a significant positive correlation with general health, pain, social function and role of physical health (Table 4.).

We examined the relationship between SF-36 HRQoL dimensions and LBPKQ scores and the found positive significant correlation only in general health status (R square: 0.17, F = 24.16, *p* < 0.01), Beta constant: 56.95, Beta LBPKQ: 0.58 *p* = 0.02) adjusting for functional status and socio-demographic parameters (age, gender, education, place of living, work type, RM).

## Discussion

CnsLBP is a notable health problem of modern societies in addition its symptoms result in the significant decrease of HRQoL in patients’ lives, more or less corresponding to socio-demographic parameters and LBP specific knowledge [31].

Several studies have proven that SF-36 questionnaire, used in the present research, is suitable for examining HRQoL of nscLBP patients: Kogure et al. [32] examined the efficiency of Arthrokinematic Approach-Hakata treatment; Tavafian et al. [33] analyzed in their research the quality of life of a LBP group participating in a Back School and a clinical treatment; Buynak et al. [34] examined the quality of life of three LBP groups, two of them receiving two kinds of opioid treatment and the third one receiving placebo; Adorno et al. [30] in their research subjected 30 patients to isostretching and global postural re-education interventions and examined the quality of life of the participants of the two treatments using SF-36 questionnaire. All of the above mentioned as well as the present research confirm the fact that the quantitative method may be adequate when examining the quality of life of nscLBP patients. Regarding the results of SF-36 dimensions the following was shown in the research of Kogure et al.: GH: 38.0 ± 8,1, BP:31.8 ± 6.7, SF: 33.4 ± 12.8, MH: 41.2 ± 8.9, VT: 39.6 ± 8.78, RE: 35.0 ± 12.5, RF: 28.9 ± 13.3, PF: 30.1 ± 14.0 [35], in the study of Tavafian et al.: GH:41.7 ± 22.2, BP: 42.6 ± 25.3, SF: 62.5 ± 29.8, MH: 47.8 ± 23.5, VT: 48.9 ± 21.6, RE: 32.7 ± 40.4, RP:31.7 ± 35.0, PF:52.5 ± 20.2 [32]. In comparison, the results found in the present research account for more favourable HRQoL, which may be caused by the fact that the set of patients taking part in studies specifying mostly interventions have a more serious nscLBP syndrome. Furthermore, the results of the RM questionnaire also confirm these findings (4.81 ± 4.59).

The HRQoL results found in our research proved to be significantly lower than normal values of the healthy population, except for Social Functioning (SF) dimension that although fell behind the mean value of the healthy population by a few score points, it did not differ significantly among nscLBP patients. Therefore, on the whole it can be stated that regarding social function the examined patients’ judgement on social function does not differ from the one of healthy population [27].

In our survey, the participants’ low back pain prevention knowledge was between 6.20–8.39 points. We also found similar results in international literature Maciel et al. [24] measured 46-year-old participants who did not receive a spine prevention education program, their point was 8.6 points. In the King of Saud University survey, 40-year-old participants who did not receive spine prevention education achieved 9 points [35].

Patients who participated in education and back school programs reached 16–22.2 points [24, 26]. The disease-specific knowledge of health workers was 19.1–19.2 points [26, 36] (Table 5).

**Table 5:** Systematic summary of disease-specific knowledge

Author/Year of publication	Place of the research	Sample (N)	LBPKQ sum value
**Maciel et al 2009** [24]	Federal University of Sao Paulo	30 46 years no education	8.6
**Awwad et al 2016** [35]	King Saud University	153 40 years no education	9.0
**Kovács-Babócsay et al 2019** [26]	University of Pécs, Hungary	54 42 years no education	12.3
**Author/Year of publication**	**Place of the research**	**Sample (N)**	**LBPKQ sum value**
**Maciel et al 2009** [24]	Federal University of Sao Paulo	30 46 years back school program- education	16.0
**Kovács-Babócsay et al 2019** [26]	University of Pécs, Hungary	54 42 years back school program- education	22.2
**Author/Year of publication**	**Place of the research**	**Sample (N)**	**LBPKQ sum value**
**Morimoto et al 2018** [36]	Hospital Sao Paulo - Universidade	60 31.7 years health care workers	19.2
**Kovács-Babócsay et al 2019** [26]	University of Pécs, Hungary	55 32 years health care workers	19.1

In our research the LBPKQ was showed a significant positive correlation with general health, pain, social function and role of physical health, which fact has important role in planning the future interventions for cnsLBP patients.

While examining the quality of life of patients bearing nscLBP syndrome our research has proven that chronic pain has a notable effect on patients’ quality of life. The longer nscLBP syndrome has been present in patients’ lives, the less favourable their quality of life is. The disease accounts for greater and greater measures of restraint regarding functional, bodily and mental health. Similar results were found when comparing RMDQ and SF-36 results: the greater the level of pain and restraint is, the more unfavourable the patients’ bodily and mental health is, furthermore the more unfavourable social function turns into. In our research patients evaluated the functional status of their spine 4.81 ± 4.59 through RMDQ. The functional status of the spine of nscLBP patients participating in international studies resulted diverse. On the whole it can be stated that studies specifying interventions are characterized by higher RMDQ values while quantitative research as the present one found lower values (Table 6) [37–40].

**Table 6:** Results of RMDQ in different international researches

Author/Year of publication	Place of the research	Sample size (N)	RMDQ sum value (standard deviation)
**van Helvoirt et al 2014** [37]	Netherlands	69	7.00–15.40
**Ghadyani et al 2016** [38]	Iran	136	5.95–6.37
**Takahashi et al 2016** [39]	Japan	102	6.68(1.57)-9.52(1.60)

According to the regression analysis our finding is that the RMDQ sum value is strongly and significantly associated with all of the 8 dimensions of HRQoL, which furthermore attests that functional drawbacks originating from the disease have an impact on all fields of HRQoL.

Among socio-demographic determinants, the significant effect of sitting for more than 30 min has been proven for the most dimensions followed by age and gender. Hence, sedentary work, the rise of age or belonging to female gender account for the highest risk on the decrease of quality of life among acLBP patients. Sedentary work accounts for a higher risk of a decrease in quality of life also in comparison with hard physical work. As it is also confirmed by our research that women, among whom the evolution of nscLBP is more frequent, evaluate their quality of life more unfavourably than men, therefore the disease means a greater burden for them, what calls for particular attention during treatment. (HRQoL was proved to be lower among women regarding BP, SF, RE, PF dimensions). The rise of age results in the decrease of HRQoL considering GH, BP, RP, PF dimensions. In the sample of the present research the elderly age-group is not represented, only the middle-aged and the aging along with the young, which fact must be taken into consideration when examining and evaluating age.

According to further findings among nscLBP patients possessing qualifications in higher education general health and physical functioning are more favourable in comparison with patients with lower education that may be explained by the assumption that people with higher education devote more attention and apply a wider range of treatment options, resulting in a higher quality of life. Moreover, low education has a significantly negative effect on social and mental health as well that reinforces nscLBP patients’ exclusion and the accumulation of further negative effects [5–7].

Regarding marital status, confirming data coming from literature, divorced and single respondents replied with less favourable values in case of dimensions concerning mental health, that proves the health preserving and improving effect, also in the case of chronic diseases, of cohabitation and marriage [9] (Table 4).

### Limitations

However, there are some important limitations of our research that should be mentioned in the present study. Firstly, our sample is not representative, the ratio of city residents is high while one of people with low education is low. Also, the sample was recruited exclusively from people coming from the Southern Transdanubia, therefore it cannot be generalized to the whole Hungarian population – conclusions in that concern can be drawn only when considering the mentioned limitation. Secondly, the HRQoL and RMDQ were measured using self-administered questionnaires, which could give bias.

## Conclusions

The present study confirmed that SF-36 is reliable to measure the HRQoL of nscLBP patients, furthermore, the patients’ HRQoL was significantly lower than the healthy populations. The LBP specific knowledge was significantly correlated with HRQoL. The age, gender – women, sitting (job) was negatively associated with most of the physical and mental HRQOL, and higher education or paid job were positively associated with physical or social HRQOL. Finally, if the marital status is divorced or single, it was negatively associated with mental HRQoL. According to these significant socio-demographic determinants an even more specific and well-aimed intervention can be framed in order to improve the HRQOL of this population.

## Data Availability

The dataset supporting the conclusions of this article is available from the corresponding author on reasonable request.

## Abbreviations

(SF-36): Short Form Health Survey
(LBPK): Low back pain knowledge
(RM): Roland Morris
(cnsLBP): Chronic non-specific low back pain syndrome
(LBP): Low back pain
(RMDQ): Roland Morris Disability Questionnaire
(PF): physical functioning
(RP): role limitations due to physical problems
(BP): bodily pain
(GH): general health
(VT): vitality
(SF): social functioning
(RE): role limitations due to emotional problems
(MH): and mental health
